# Do decision support systems influence variation in prescription?

**DOI:** 10.1186/1472-6963-9-20

**Published:** 2009-01-30

**Authors:** Judith D de Jong, Peter P Groenewegen, Peter Spreeuwenberg, Gert P Westert, Dinny H de Bakker

**Affiliations:** 1 NIVEL-Netherlands Institute for Health Services Research, PO Box 1568, 3500 BN Utrecht, the Netherlands; 2 Utrecht University, Department of Sociology and Department of Human Geography, Utrecht, the Netherlands; 3 RIVM-National Institute of Public Health and the Environment, PO Box 1, 3720 BA Bilthoven, the Netherlands; 4 Tilburg University, Faculty of Social and Behavioral Sciences Social Cultural Sciences, Tilburg, the Netherlands

## Abstract

**Background:**

Translating scientific evidence into daily practice is problematic. All kinds of intervention strategies, using educational and/or directive strategies, aimed at modifying behavior, have evolved, but have been found only partially successful. In this article the focus is on (computerized) decision support systems (DSSs). DSSs intervene in physicians' daily routine, as opposed to interventions that aim at influencing knowledge in order to change behavior. We examined whether general practitioners (GPs) are prescribing in accordance with the advice given by the DSS and whether there is less variation in prescription when the DSS is used.

**Methods:**

Data were used from the Second Dutch National Survey of General Practice (DNSGP2), collected in 2001. A total of 82 diagnoses, 749811 contacts, 133 physicians, and 85 practices was included in the analyses. GPs using the DSS daily were compared to GPs who do not use the DSS. Multilevel analyses were used to analyse the data. Two outcome measures were chosen: whether prescription was in accordance with the advice of the DSS or not, and a measure of concentration, the Herfindahl-Hirschman Index (HHI).

**Results:**

GPs who use the DSS daily prescribe more according to the advice given in the DSS than GPs who do not use the DSS. Contradictory to our expectation there was no significant difference between the HHIs for both groups: variation in prescription was comparable.

**Conclusion:**

We studied the use of a DSS for drug prescribing in general practice in the Netherlands. The DSS is based on guidelines developed by the Dutch College of General Practitioners and implemented in the Electronic Medical Systems of the GPs. GPs using the DSS more often prescribe in accordance with the advice given in the DSS compared to GPs not using the DSS. This finding, however, did not mean that variation is lower; variation is the same for GPs using and for GPs not using a DSS. Implications of the study are that DSSs can be used to implement guidelines, but that it should not be expected that variation is limited.

## Background

According to Grol and Grimshaw [1]: *"One of the most consistent findings in research of health services is the gap between evidence and practice. Results of studies in the USA and the Netherlands suggest that about 30–40% of patients do not receive care according to present scientific evidence, and about 20–25% of care provided is not needed or is potentially harmful"*. These are causes of unwanted variations in health service provision. Introducing evidence and clinical guidelines into daily practice is problematic. All kinds of intervention strategies, using educational and/or directive strategies, aimed at modifying behavior, have been developed, but found only partially successful [e.g. [1-3]].

Although different treatments can be effective, and innovations are not likely to occur when there is no variation, the existence of variation will have an effect on the profession of medicine. Physicians will have to explain why there is variation. Meaning that they will have to explain why similar patients with the same diagnosis are treated differently. Policy makers and third party payers will get involved as they might be convinced that health care expenses can be limited when all physicians choose the most cost-effective treatment. Variation might give patients a choice in the treatment they prefer, and it might help in finding better treatments, but as long as there are questions about the justifiability of variation physicians will have to deal with it, limit or explain it, or otherwise insurers or the government probably will.

This article focuses on computerized decision support systems (DSSs). The use of DSSs is a way to improve physicians' performance [4]. Computerized systems rationalize medical practice, by implementing evidence into medical practice, and in doing so decrease variation in medical practice [5,6]. They are meant to improve the capacity of physicians to make better decisions whilst the complex problems physicians deal with surpass their cognitive capacity [5]. Computerized systems intervene in physicians' daily routine, as opposed to interventions that aim at influencing knowledge in order to change behavior. Intervening in physicians' daily routine was found to be effective in changing behavior for instance in blood test ordering [7].

In the Netherlands a DSS for prescribing drugs was introduced for general practitioners (GPs) in 1998. The DSS was introduced to implement professional guidelines regarding the prescription of drugs. Prescription of drugs has great importance in modern health care; the development of new and effective drugs has contributed to the increase of the health status in the OECD countries [8]. In modern health care, appropriate prescribing of new and expensive drugs is a big challenge as the number of available drugs is increasing, making choice more complex.

Prescription of drugs is influenced by the pharmaceutical industry [e.g. [9]], the professional environment [e.g. [2]], physicians' and patients' habits [e.g. [10,11]]; all factors that are not directly related to treatment outcome. Denig [12] showed that physicians choose from a limited evoked set of drugs which comes up in their minds, given the health problem of the patient. This evoked set is influenced by advertising, and (continuing) education. More than 70% of drugs prescribed are drugs from the evoked set [13]. This 70% is 'top of the mind' drugs, meaning that, given the diagnosis, these drugs come to the mind directly, based on habits. Physicians do not consider all possible treatment options, but chose from approximately two to five different options [12]. In order to change the drugs physicians prescribe, the evoked set of physicians has to be changed, or its role in decision making should be changed. The evoked set should become less important or should even be avoided when a change in prescribing behavior is necessary. That is where DSSs come in. Instead of relying on the evoked set of drugs for physicians to choose one in a specific situation, the DSS proposes one or more drugs of preference, based on characteristics of the patient and professional guidelines. The introduction of DSSs is expected to result in more rational prescribing and less unexplained and illegitimate variation in prescribing between physicians [12,14-16].

Educational interventions and giving feedback as methods to enhance evidence based practice rely on changing the evoked set in the minds of physicians. DSSs, however, directly affect physicians' routines. The strategy of changing physicians' routines has shown to be effective in for instance blood test ordering. Zaat et al. [17] found that by simply changing the application form, the amount of unnecessary test ordering was drastically decreased. After changing the form back into its original format the physicians showed their old test ordering behavior.

DSSs can be useful tools in medical practice; they can assist physicians in making evidence based decisions and can therefore improve the quality of care. Quality of care will only improve with the use of DSSs if the recommendations are evidence based and the DSS is actually used as intended. DSSs do therefore not always improve clinical practice. Hunt et al. [18] showed in their systematic review that most systems significantly improved clinical practice, but some (34%) did not. Lobach and Hammond [19] found in their study on clinical practice in the USA that DSSs improved compliance with care standards. Shea et al. [20] found evidence from a meta-analysis of randomized controlled studies that supports the effectiveness of data-driven computer-based reminder systems to improve prevention services in the ambulatory care setting. Johnston et al. [21] concluded from their review that recommendations from DSSs can improve compliance with guidelines for preventive and acute care. Improvement of guideline adherence was also shown by Shiffman et al. [22] in their systematic review on the effectiveness of computer-based guideline implementation systems. In a systematic review on the effects of DSSs on physician performance and patient outcomes it was concluded that DSSs can enhance clinical performance for drug dosing, preventive care, and other aspects of medical care, but not convincingly for making the diagnosis [18]. Furthermore, they concluded that the effects on patient outcomes have been insufficiently studied. A study by Ramnarayan et al. [23] suggests a promising role for DSSs in the reduction of diagnostic error. DSSs result in improved clinical practice if support is provided automatically as part of physicians' workflow, support is delivered at the time and location of decision making, actionable recommendations are provided and support is computer based [6].

DSSs can influence physicians' behavior and at the same time influence variation in prescribing between physicians. Generally, variation is reduced by shared normative systems, rules and common frameworks of meaning [24]. That is, variation decreases when people adhere to the same norms and rules, or make use of the same frameworks of meaning. DSSs can be considered frameworks of meaning, people do not have to be aware of what a DSS does when they use it and DSSs do not work via behavioral confirmation or sanctioning as norms and rules do. As such they can decrease variation amongst those who make use of the DSS.

In this article we will study a DSS which is used by GPs in the Netherlands as a tool to give advice on prescription when the diagnosis is given. The DSS proposes a prescription, given the diagnosis of the patient, taking into account age, sex and co-morbidity. As mentioned before, four features are associated with the ability to improve clinical practice, it is part of the workflow, support is delivered at the time and location of decision making, actionable recommendations are provided and it is computer based [6]. The DSS for GPs in the Netherlands meets these four features [25]. In the advice patient characteristics, like age, sex, co-morbidities and other drugs prescribed are taken into account. The DSS is integrated in the Electronic Medical Systems (EMS) of the GPs. The advice given is derived from professional-guidelines. These guidelines are developed by the Dutch College of General Practitioners (NHG) and are widely accepted [26]. Wolters et al. [27] studied the use of the DSS by GPs in the Netherlands. They found that having access to the DSS increased from 20% in 1999 to 71% of GPs in 2001, and daily use from 11% to 40%.

In this article we will examine the influence of computerized decision support on prescribing by GPs in the Netherlands. Two questions will be addressed.

*1 *'*Do GPs who use the DSS on a regular basis prescribe more often in accordance with the advice given by the DSS?'*

2 'Is there less variation in prescription among GPs who use the DSS on a regular basis compared to those who do not?'

### Hypotheses

An effective method for changing physicians behavior, or implementing new techniques, is to make them save time if they change or comply [28]. An effective DSS minimizes the effort required by physicians to receive and act on the recommendations [6]. DSSs in the Netherlands give GPs information at the time a decision has to be made. Thus, GPs do not have to recall information from the mind, and can more easily prescribe according to professional guidelines, even without having to make a conscious choice. *It is expected that those who use the DSS, prescribe according to the guidelines that are incorporated in the DSS*.

The more GPs use the DSS, the more their prescriptions will be in accordance with the advice given in the DSS. If GPs use the DSS there will be less variation in prescription. *It is expected that there is less variation in prescribing between GPs using the DSS compared to GPs not using the DSS*.

## Methods

### Data

Data were used from the Second Dutch National Survey of General Practice (DNSGP2), collected in 2001. Data were collected on contacts, patients, GPs and practices. For a description of the data-collection we refer to Westert et al. [29]. DNSGP2 is used for many different research questions and analyses. The basis of DNSGP2 was an extraction of the electronic medical records from 103 general practices during one year. The electronic medical records contained the Anatomical Therapeutic Chemical (ATC)-coded prescription data [30]. DSS use was assessed through a questionnaire filled in by 191 GPs working in the 103 practices in March 2001, 188 questionnaires could be used. The response was 96%. In the questionnaire it was asked if the GPs had a DSS and how many times the DSS was used. The questionnaire was not designed for this specific article and consequently the GPs were not aware of this study. The current study is a secondary analysis of the DNSGP2 data base. The total DNSGP2 population can be found in Table 1[31]. The study was carried out in keeping with Dutch legislation on privacy. Compliance with privacy regulations was approved by the Dutch Data Protection Authority. According to Dutch legislation, neither obtaining informed consent nor approval by a medical ethics committee was obligatory for this study

**Table 1:** Selection of GPs used in this article, the study population of DNSGP2 and the total population of Dutch GPs at the time of data collection.

	*GPs included in this article from DNSGP2 (%)*	*Study population DNSGP2 (%)*	*Total GP population in the Netherlands in 2001 (%)*
Sex:			
Male	78	73	74
Female	22	27	26
			
Age:			
<35	1	4	6
35–39	15	15	15
40–44	15	18	20
45–49	32	30	25
50–54	25	25	22
55–59	12	8	10
60+	1	1	2
			
Type of practice:			
Single handed	51	31	43
Shared	24	28	33
Group/Health centre	26	42	25

### Method

The DSS includes an advice for 172 diagnoses. These diagnoses were included in our analyses if in at least 1,250 contacts a prescription was given for this diagnosis. A minimum was set to exclude diagnoses that hardly appear in GPs practices. The minimum was set at 1,250 contacts for computational restrictions. A total of 82 diagnoses were included in the analyses. Contacts were included only if type of medicine (ATC-5) and diagnosis (ICPC coded) were known. All participating GPs have had a coding training at inclusion in DNSGP2. Because of missing diagnoses sixteen practices were excluded. A description of the GPs in our analysis compared to the total DNSGP2 study group as well as to the total population in the Netherlands can be found in Table 1. There is an overrepresentation of single handed practices in our population. A total of 749,811 contacts, 133 physicians, and 85 practices were thus included in the analyses (Table 2).

**Table 2:** Description of included data (absolute numbers)

	Included data*	*DSS daily users*	*DSS non users/owners*
Number of practices	85	29	33
Number of physicians	133	44	43
Number of patients	749811	251587	242786
Number of diagnoses	82	82	82
			
Type of practice:			
Group	22	10	9
Dispensing (pharmacy included)	9	3	1
Dispensing (with pharmacy)	1	0	0
Not dispensing (no pharmacy)	73	26	32
Missing	2	0	0
TOTAL	85	29	33
			
EMS:			
Microhis	23	5	16
Promedico	29	14	5
Elias	22	7	10
Arcos	11	3	2
TOTAL	85	29	33
			
Number of physicians per practice (mean (st.dev) [range])	1.6 (1.1) [1–5]	1.5 (1.1) [1–5]	1.3 (0.7) [1–4]
Number of different drugs per diagnosis in DSS (mean (st.dev) [range]	4.0 (2.4) [1–11]	4.0 (2.4) [1–11]	4.0 (2.4) [1–11]
Number of different drugs prescribed per physician per diagnosis (mean (st.dev) [range])	7.0 (6.7) [1–156]	6.7 (6.1) [1–66]	7.2 (6.9) [1–77]

*selected based on whether diagnoses are in the DSS

For this study two extremes were created: those GPs who use the DSS daily and those GPs who do not use the DSS at all. The latter group both includes GPs who do not have a DSS and GPs who do have a DSS but do not use it. An important difference between both groups is in the Electronic Medical System they use. We have controlled for this difference in the analyses.

Specifics in the methods differ between the two questions addressed in this article. Therefore, the specific methods will be described for each question separately.

#### Are GPs prescribing in accordance with the advice given by the information system?

##### Dependent variable

As dependent variable a measure was used that indicated whether a prescription was in accordance with DSS or not. For each diagnosis (ICPC coded) a list of prescriptions (ATC-5 coded) advised in the DSS was used [25]. These proposed prescriptions were compared with the actual prescriptions for patients. If prescriptions for patients were similar to the prescription in the list it was in accordance with the DSS, if it was different it was not in accordance with the DSS. The comparison was done for both GPs using the DSS and for GPs not using the DSS.

##### Model

Multilevel analyses were used to take into account the structure of the data: contacts are nested within GPs and GPs are nested within practices [32-34]. The model therefore consists of three levels; the contact, the GP and the practice level. Because the dependent variable was dichotomous, a multilevel logistic regression was performed (see appendix 1 for the full model). The percentage of contacts in which patients receive prescriptions in accordance with the DSS was computed, taking the diagnosis into account and correcting for the specific EMS, practice type, and the number of GPs in a practice. Besides, a variable was included indicating whether or not a practice was dispensing for all or part of the population. The variable was included because medication prescribed by medical specialists was also included in the data collection for GPs in a dispensing practice, but are not recognized as such. Hence, in these practices part of the prescribing decisions might have been made by medical specialists. For the model to have interpretable meaning, variables were centered around their means. Diagnoses were not only included in the fixed part of the model, but also in the random part of the model. The latter was done because the observations are not independent; different physicians face patients with the same diagnoses and hence the same advice of the DSS which has an effect on what is prescribed.

#### Is there less variation in prescription when the information system is used?

##### Dependent variable

As dependent variable to measure variation in prescribing a measure of concentration was used, the Herfindahl-Hirschman Index (HHI) [35,36]. This measure was based on whether the prescriptions for a given diagnosis were distributed over a large number of different drugs or only one or a few drugs. The kind of drug was identified based on the ATC-5 code. The HHI was measured as Σ(a/b)^2^; where *a *is the number of times a specific drug was prescribed per diagnosis per GP and *b *is the total number of times any drug is prescribed for this diagnosis per GP. The HHI was measured for each drug prescribed per diagnosis per GP and these values were summed for all drugs prescribed per diagnosis per GP. The range of this index goes from a low point of 1 divided by the number of drugs prescribed per diagnosis to a maximum of 1. A low index means that all drugs are equally often prescribed while 1 means that there is only one drug prescribed. The higher the concentration the less variation in drug prescription there is. The HHI was multiplied by 100 for ease of interpretation of the coefficients.

In contrast to the previous model, the HHI, the concentration index, is not a characteristic of a single patient contact but of the aggregate of patient contacts with the same diagnosis. It is measured per diagnosis per GP.

##### Model

The data are hierarchically structured and therefore multilevel models are the appropriate statistical approach [32-34]. The model consists of three levels; diagnoses, GPs and practices. Since different GPs face patients with similar diagnoses, again a model, which allows for dependence between observations, was chosen (see appendix 2 for the full model).

To account for differences in patient population between GPs the mean age and sex of the patient population of a GP were initially included. These variables did only have a small effect and were excluded from the final analyses. Corrections were made for the different diagnoses by including all diagnoses in the random part of the model. They were, as opposed to the previous analysis, not included in the fixed model because the HHI was measured per diagnosis. As mentioned before, the HHI was measured per GP per diagnosis and therefore each diagnosis occurred only once for each GP. Furthermore, the specific EMS, practice type, the number of GPs in a practice, and the number of different drugs suggested in the DSS per diagnosis were included. A variable was included indicating whether or not a practice was dispensing for all or part of the population. As in the previous model, all variables were centered to give the model interpretable meaning.

## Results

### Are GPs prescribing in accordance with the advice given by the information system?

In line with our expectation GPs who use the DSS daily prescribe more according to the advice given in the DSS than GPs who do not use the DSS (Table 3). Still, prescription by GPs who do not use the DSS is in line with advices given in the DSS in 75% of all prescriptions, against 89% for daily users. In appendix 3 the total model is presented. The fixed effects show that there are differences between diagnoses in whether GPs prescribe in accordance with the advice given in the DSS.

**Table 3:** Proportion of prescriptions in accordance with DSS, corrected for EMS, type of practice, dispensing practice, number of GPs in a practice (full model in appendix 3)

	*in accordance with DSS (st. error)*	*Difference*
DSS daily users	0.89 (0.06)	
DSS non users/havers	0.75 (0.05)	0.14 (p = 0.04)

### Is there less variation in prescription when the information system is used?

Contradictory to our expectation there is no significant difference between the HHIs, the indicator we used to measure variation, for GPs using the DSS daily and GPs who do not own or use the DSS (Table 4). Apparently the variation in prescription is comparable for both groups. In appendix 4 the total model is presented. The fixed part of the model shows a relatively strong effect of the number of different drugs advised in the DSS. If this number is larger there is more variation in prescribing. From the random part we learn that the HHI shows differences between diagnoses. Comparing groups of diagnoses, variation in HHI is relatively low in diagnoses related to the skin and relatively high in the diagnoses from the chapter 'general' of the ICPC, the first five diagnoses of the list.

**Table 4:** Variation in prescribing, as measured by HHI, corrected for EMS, type of practice, dispensing practice, number of GPs in a practice, number of different drugs advised in the DSS (full model in appendix 4)

	*HHI (st. error)*	*Difference*
DSS daily users	40.3 (1.2)	
DSS non users/havers	41.4 (1.3)	1.1 (p = 0.3)

## Discussion

GPs using a DSS are prescribing in accordance with the advice given in the DSS more than GPs not using a DSS. Still, variation is the same for GPs using a DSS and for GPs not using a DSS.

The DSS for prescribing by GPs in the Netherlands incorporates the professional guidelines of the Dutch College of General Practitioners. Therefore, the DSS can be seen as a way to implement guidelines. Guidelines and their incorporation in a DSS are part of a general trend towards rationalization of medicine in the Western world and one of the general implications of rationalization processes is a decrease in variation. The use of DSSs was hypothesized to decrease variation between physicians, because physicians who use a DSS that is based on the same professional guidelines make use of the same cognitive framework.

Although GPs are using the DSS and thus the same cognitive framework, variation did not decrease. How can we explain this finding? DSSs give recommendations for prescribing certain drugs. The DSS for prescribing by GPs that we studied advises several different drugs or recommends a stepwise treatment starting with one type of drug and changing that type of drug later on when necessary. We were not able to take stepwise use of different drugs into account in our definition of conformity to the advice of the DSS. As a consequence, variation can be generated. GPs not using the DSS prescribe from their evoked set and probably do not use a stepwise treatment strategy.

Although variation does not necessarily mean that some patients receive bad quality of care, it does raise questions related to effectiveness, efficiency and equity [37]. Evidence on medical practice variations implicates that there might be inappropriate servicing, waste of resources and maybe even harm to patients [38].

In the Netherlands the advice given in the DSS is derived from professional guidelines, developed by the Dutch College of General Practice. The formularies correspond to the NHG-guidelines for 65–70% [14]. The NHG-guidelines both reflect what is common in the profession and what should become common [39,40]. Only part of the NHG-guidelines published between 1989 and 2000 are evidence-based. They are mostly based on consensus between members of the study groups that are involved in the development of the guidelines [41]. Of 130 recommendations in 28 NHG-guidelines published between 1993 and 1997, 44% was evidence-based [42]. Scientific evidence is developing continuously and guidelines should therefore be changed according to the latest evidence on a regular basis. GPs can be ahead of changes in the guideline and already use new evidence before the guideline is updated [43]. Guidelines reflecting common practice, and GPs being ahead of guidelines can explain that GPs not using the DSS prescribe in accordance with the advice. The fact that we did not find a difference in variation between daily users and non-users of the DSS indicates that the introduction of the DSS did not lead to simple cookbook medicine.

This article does not question whether or not the professional guidelines, incorporated in the DSS, are adequate and whether or not prescribing quality is better for the users of the DSS. These are different questions from the one discussed in this paper and would require a guideline by guideline analysis of evidence and data. In our article we only tested whether or not over a large domain of diagnoses, there is indeed more conformity to the advice of the DSS among daily users than among non-users.

One of the limitations of this study is that we do not know whether there has been a change in the behavior of GPs when they started using the DSS. GPs using the DSS were compared to GPs not using the DSS in the same time period. We could not perform a longitudinal analysis which would make it possible to detect a change in behavior. From the analysis performed, we conclude that GPs using the DSS prescribe according to it and thus comply with professional guidelines more than GPs not using the DSS. It is assumed that GPs change their prescribing behavior when they start using the DSS, but it is possible that they already did prescribe in accordance with the advice. Another limitation is that coding the diagnosis is a condition to use the DSS. We used only data from practices coding the diagnoses in the ICPC-system. We needed these codes to be able to construct our dependent variable that indicates conformity to the advice, given by the DSS. It is possible that the non-users of the DSS that nevertheless use the ICPC-coding in their EMR are a positive selection.

We assume that the coded diagnosis reflects what GPs think is the diagnosis at the time they enter its code in the system. It is this diagnosis that steers their prescription decision, irrespective of whether or not this diagnosis was correct or incorrect.

Although the questions answered in this article are fairly simple, the analyses were complicated. For both questions it was necessary to take the diagnoses into account. Furthermore, variation in prescription is not measured easily. To overcome the first difficulty, we could have chosen to examine specific diagnoses. Analyzing every diagnosis separately and aggregating the results over all diagnoses would ignore the fact that physicians' prescribing decisions in one diagnosis are correlated to the same physicians' prescribing decisions in another diagnosis. Moreover, our model structure takes into account the fact that prescribing decisions of different physicians for the same diagnosis will be correlated as well. Moreover, we were testing a general hypothesis and having separate outcomes for each (group of) diagnosis would ignore the correlation of prescribing decisions. To take the specific diagnoses into account and the fact that the same diagnosis is encountered by different GPs, we used a model, taking into account that the same diagnosis can 'belong' to different GPs. The statistical model we developed has the additional advantage that it is able to identify (groups of) diagnoses with relatively low and high conformity to the DSS' advice, taking the data structure into account.

The second difficulty, how to measure variation in prescription, had several possible solutions. Counting the range, the number of different drugs prescribed, was one possibility [44]. Disadvantage of the range, however, is its insensitivity to the number of times a drug is prescribed. For example, the range is two if both drugs are each ten times prescribed, but also when one drug is ten times prescribed and the other is prescribed a hundred times. Measures of concentration are sensitive for the number of times a drug is prescribed. Therefore, the Herfindahl-Hirschman Index (HHI), which is a measure of concentration, was used; the higher the HHI the less different drugs are prescribed.

This study implicates that physicians decision making regarding prescription can be influenced with computerized decision aids. Although we only studied the effect of the DSS for drug prescribing in general practice in the Netherlands, the result we found is in line with the results of international reviews. To improve the quality of prescribing these systems should be up-to-date and the advice should be evidence based. However, the regular use of a DSS did not result in lower practice variation.

## Conclusion

This study demonstrates that GPs using a DSS are prescribing in accordance with the advice given in the DSS more than GPs not using a DSS. This implicates that compliance with guidelines improves when the DSS is used. This finding, however, did not mean that variation is lower; variation is the same for GPs using and for GPs not using a DSS. Implications of the study are that DSSs can be used to implement guidelines, but that it should not be expected that variation is limited. As DSSs can influence prescription, it is important to make explicit who is developing the DSS and for what reason.

## Competing interests

The authors declare that they have no competing interests.

## Authors' contributions

JdJ performed the statistical analyses, drafted the manuscript and contributed to all other aspects of the study. PG participated in the design of the study, the critical revision of the manuscript and its supervision. PS participated in the design of the study and analyses of the data. GW contributed to the acquisition of data and participated in the revision of the manuscript. DdB participated in the design of the study, the critical revision of the manuscript and its supervision. All authors have given final approval of the submitted manuscript.

## Appendix 1: The multilevel logistic model

*γijk*~ Binomial(*nijk*, *πijk*)

$$\gamma_{ijk}=\pi_{ijk}+e_{0ijk}x_{0}^{*}$$

logit (*πijk*) = *β*_0*jk*_*x*_0*jk *_+ *β*_1*jk*_*x*_1*jk *_+ *β*_2_*x*_2*k *_+ *β*_3_*x*_3*k *_+ *β*_4_*x*_4*k *_+ *β*_5_*x*_5*k *_+ *β*_6_*x*_6*k *_+ *β*_7_*x*_7*k *_+ *β*_8_*x*_8*k *_+ *β*_9_*x*_9*k *_+ *β*_10_*x*_10*ijk *_+ ..... + *β*_90_*x*_90*ijk *_+ *e*_91*i*_*x*_91*ijk *_+ ...... + *e*_172*i*_*x*_172*ijk*_

*β*0*jk *= *β*0 + *v*_0*k *_+ *u*_0*jk*_

*β*1*jk *= *β*1 + *v*_1*k *_+ *u*_1*jk*_

$$\left[\begin{array}{c} v_{0k}\\ v_{1k} \end{array}\right]\sim N(0,\Omega_{v}):\Omega_{v}=\left[\begin{array}{cc} \sigma_{v0}^{2} & \\ 0 & \sigma_{v1}^{2} \end{array}\right]$$

$$\left[\begin{array}{c} u_{0jk}\\ u_{1jk} \end{array}\right]\sim N(0,\Omega_{u}):\Omega_{u}=\left[\begin{array}{cc} \sigma_{u0}^{2} & \\ 0 & \sigma_{u1}^{2} \end{array}\right]$$

$$\left[\begin{array}{l} e_{91ijk}\\ \begin{array}{c} .......\\ e_{172ijk} \end{array} \end{array}\right]\sim N(0,\Omega_{e}):\Omega_{e}=\left[\begin{array}{ll} \sigma_{e91}^{2} & \\ 0............... & \\ 0......................... & \sigma_{e172}^{2} \end{array}\right]$$

*γijk *= dependent variable (conform DSS or not, dichotomous)

*i *= 'patient populations' (an identifier for each diagnosis per physician)

*j *= physicians

*k *= practices

*β*0*jk *= mean propotion conform DSS, per physician (daily users)

*β*1*jk *= mean proportion conform DSS, per physician (non users/havers)

*x*_2 _= EMR1, per practice (centred)

*x*_3 _= EMR2, per practice (centred)

*x*_4 _= EMR3, per practice (centred)

*x*_5 _= Duo practice, per practice (centred)

*x*_6 _= Group practice, per practice (centred)

*x*_7 _= Health centre, per practice (centred)

*x*_8 _= Dispensing practice, per practice (centred)

*x*_9 _= number of GPs, per practice (centred)

*x*_10 _..........*x*_90 _= diagnosis (centred)

*x*_91 _..........*x*_172 _= diagnosis

v = level -three variance (practice level)

u = level -two variance (physician level)

e = measurement error per diagnosis

## Appendix 2: The multilevel model

*γ*_*ijk*_~ *N*(*XB*,Ω)

*γi*_*jk *_= *β*_0*jk*_*x*_0*jk *_+ *β*_1*jk*_*x*_1*jk *_+ *β*_2_*x*_2*k *_+ *β*_3_*x*_3*k *_+ *β*_4_*x*_4*k *_+ *β*_5_*x*_5*k *_+ *β*_6_*x*_6*k *_+ *β*_7_*x*_7*k *_+ *β*_8_*x*_8*k *_+ *β*_9_*x*_9*k *_+ *β*_10*i*_*x*_10*ijk *_+ ..... + *e*_91*i*_*x*_91*ijk*_

*β*0*jk *= *β*0 + *v*_0*k *_+ *u*_0*jk*_

*β*1*jk *= *β*1 + *v*_1*k *_+ *u*_1*jk*_

$$\left[\begin{array}{c} v_{0k}\\ v_{1k} \end{array}\right]\sim N(0,\Omega_{v}):\Omega_{v}=\left[\begin{array}{cc} \sigma_{v0}^{2} & \\ 0 & \sigma_{v1}^{2} \end{array}\right]$$

$$\left[\begin{array}{c} u_{0jk}\\ u_{1jk} \end{array}\right]\sim N(0,\Omega_{u}):\Omega_{u}=\left[\begin{array}{cc} \sigma_{u0}^{2} & \\ 0 & \sigma_{u1}^{2} \end{array}\right]$$

$$\left[\begin{array}{l} e_{10ijk}\\ \begin{array}{c} .......\\ e_{91ijk} \end{array} \end{array}\right]\sim N(0,\Omega_{e}):\Omega_{e}=\left[\begin{array}{ll} \sigma_{e10}^{2} & \\ 0............... & \\ 0......................... & \sigma_{e91}^{2} \end{array}\right]$$

*γijk *= dependent variable (HHI, per diagnosis per physician)

*i *= 'patient populations' (an identifier for each diagnosis per physician)

*j *= physicians

*k *= practices

*β*0*jk *= mean HHI and variance parameters, per physician (daily users)

*β*1*jk *= mean HHI and variance parameters, per physician (non users/havers)

*x*_2 _= EMR1, per practice (centred)

*x*_3 _= EMR2, per practice (centred)

*x*_4 _= EMR3, per practice (centred)

*x*_5 _= Duo practice, per practice (centred)

*x*_6 _= Group practice, per practice (centred)

*x*_7 _= Health centre, per practice (centred)

*x*_8 _= Dispensing practice, per practice (centred)

*x*_9 _= number of GPs, per practice (centred)

*x*_10 _..........*x*_91 _= diagnosis

v = level -three variance (practice level)

u = level -two variance (physician level)

e = measurement error per diagnosis

Appendix 3 Proportion of prescriptions in accordance with DSS

Fixed effects     Estimate (st. error)

DSS use:

DSS daily users     0.89 (0.06)

DSS non users/havers     0.75 (0.05)

EMR:

EMR1 (centered)     0.42 (0.13)

EMR2 (centered)     0.28 (0.12)

EMR3 (centered)     0.14 (0.13)

Type of practice:

Duo practice (centered)     -0.13 (0.10)

Group practice (centered)     0.02 (0.17)

Health centre (centered)     -0.06 (0.22)

Dispensing practice (centered)     -0.27 (0.16)

Number of GPs (centered)     0.05 (0.05)

Diagnoses:

Allergy/allergic reaction nos (centered)     -0.21 (0.06)

No disease (centered)     -5.51 (0.36)

Iron deficiency anemia (centered)     1.21 (0.07)

Pernicious/folate deficiency anemia (centered)     -2.54 (0.10)

Stomach ache/stomach pain (centered)     1.37 (0.06)

Heartburn (centered)     1.40 (0.06)

Nausea (centered)     0.20 (0.07)

Diarrhea (centered)     -0.39 (0.07)

Constipation (centered)     0.60 (0.05)

Other presumed infections of digestive system (centered)     -2.07 (0.10)

Disease of esophagus (centered)     1.76 (0.06)

Disorders of stomach function/gastritis (centered)     1.43 (0.06)

Irritable bowel syndrome (centered)     -0.91 (0.05)

Anal fissure/perianal abscess (centered)     -2.24 (0.10)

Abnormal sensations of eye (centered)     0.27 (0.06)

Infectious conjunctivitis (centered)     1.07 (0.06)

Allergic conjunctivitis (centered)     0.05 (0.06)

Otitis externa (centered)     0.61 (0.05)

Acute otitis media/myringitis (centered)     0.35 (0.05)

Vertiginous syndrome/labyrinthitis/vestibulitis (centered)     0.82 (0.07)

Angina pectoris (centered)     0.55 (0.05)

Heart failure (centered)     -0.11 (0.05)

Uncomplicated hypertension (centered)     0.20 (0.04)

Hypertension with involvement target organs (centered)     -0.31 (0.05)

Transient cerebral ischemia (centered)     1.10 (0.07)

Hemorrhoids (centered)     -0.50 (0.06)

Rheumatoid arthritis and allied conditions (centered)     -0.43 (0.05)

Osteoarthritis of hip (centered)     -0.12 (0.07)

Osteoarthritis of knee (centered)     -0.19 (0.06)

Other osteoarthritis and allied conditions (centered)     -0.25 (0.07)

Osteoporosis (centered)     -0.48 (0.06)

Headache (centered)     -0.10 (0.05)

Tension headache (centered)     0.09 (0.07)

Restless legs syndrome (centered)     -1.90 (0.08)

Vertigo/dizziness (centered)     0.23 (0.06)

Parkinsonism/paralysis agitans (centered)     0.11 (0.08)

Epilepsy, all types (centered)     -3.38 (0.10)

Migraine (centered)     1.81 (0.06)

Disturbances of sleep/insomnia (centered)     1.53 (0.04)

Affective psychosis (centered)     -2.27 (0.11)

Anxiety disorder/anxiety state (centered)     0.81 (0.05)

Depressive disorder/anxiety/depression (centered)     0.42 (0.04)

Cough (centered)     -0.84 (0.04)

Sympt/complt sinus (incl. Pain) (centered)     -2.77 (0.12)

U.R.I. (head cold)/rhinitis nos (centered)     -1.80 (0.05)

Sinusitis acute/chronic (centered)     -0.38 (0.04)

Tonsillitis acute (centered)     0.52 (0.06)

Acute laryngitis/tracheitis/croup (centered)     -2.48 (0.11)

Acute bronchitis/bronchiolitis (centered)     -4.06 (0.07)

Pneumonia (centered)     -0.88 (0.06)

Chronic bronchitis/bronchiectasis (centered)     0.52 (0.06)

Emphysema/chronic obstructive pulmonary disease (centered)     0.76 (0.04)

Asthma (centered)     0.89 (0.04)

Hayfever, allergic rhinitis (centered)     0.66 (0.04)

Localised redness/erythema/rash of skin (centered)     -3.72 (0.17)

Herpes zoster (centert)     -0.13 (0.08)

Dermatophytosis (centered)     1.29 (0.05)

Moniliasis/monilia infection/candidiasis (centered)     -2.22 (0.07)

Other infectious skin dis.nec/erysipelas (centered)     -0.26 (0.06)

Impetigo (centered)     0.59 (0.07)

Seborrhoic dermatitis/other erythematous dermatoses (centered)     -0.64 (0.05)

Atopic dermatitis/eczema (centered)     0.85 (0.05)

Contact dermatitis/skin allergy (centered)     0.28 (0.04)

Psoriasis w/wo arthropathy (centered)     0.39 (0.06)

Acne (centered)     1.95 (0.07)

Urticaria (centered)     -1.03 (0.06)

Other disease skin/subcutaneous tissue (centered)     -1.77 (0.07)

Hyperthyroidism/thyrotoxicosis (centered)     0.76 (0.08)

Hypothyroidism/myxedema (centered)     2.06 (0.07)

Diabetes mellitus (centered)     0.40 (0.04)

Gout (centered)     1.21 (0.07)

Lipid metabolism disorder (centered)     1.35 (0.05)

Cystitis/other urinary infect. Non-venereal (centered)     0.68 (0.04)

Family planning/oral contraceptive (centered)     0.33 (0.04)

Menstrual pain (centered)     1.25 (0.09)

Menstruation excessive (centered)     0.78 (0.08)

Menstruation irregular/frequent (centered)     0.74 (0.09)

Menopausal sympt./complt. (centered)     -0.33 (0.05)

Urogenital candidiasis, thrush (proven/unproven) (centered)     -0.30 (0.05)

Vaginitis/vulvitis, non venereal nos (centered)     -0.77 (0.07)

Benign prostatic hypertrophy (centered)     0.48 (0.08)

Random effects     Variance (st. error)

Practice level:

DSS daily users     0.00 (0.00)

DSS non users/havers     0.03 (0.02)

GP level:

DSS daily users     0.11 (0.02)

DSS non users/havers     0.04 (0.02)

Diagnosis level:

Pain: generalised/unspecified     0.99 (0.03)

Allergy/allergic reaction nos     1.03 (0.03)

No disease     1.00 (0.05)

Iron deficiency anemia     0.95 (0.03)

Pernicious/folate deficiency anemia     0.92 (0.04)

Stomach ache/stomach pain     0.96 (0.02)

Heartburn     0.95 (0.02)

Nausea     1.11 (0.04)

Diarrhea     0.98 (0.04)

Constipation     0.99 (0.01)

Other presumed infections of digestive system     1.02 (0.05)

Disease of esophagus     0.91 (0.02)

Disorders of stomach function/gastritis     0.97 (0.02)

Irritable bowel syndrome     1.02 (0.02)

Anal fissure/perianal abscess     1.03 (0.05)

Abnormal sensations of eye     0.92 (0.03)

Infectious conjunctivitis     1.03 (0.02)

Allergic conjunctivitis     0.97 (0.03)

Otitis externa     0.97 (0.02)

Acute otitis media/myringitis     1.03 (0.02)

Vertiginous syndrome/labyrinthitis/vestibulitis     0.94 (0.03)

Angina pectoris     1.00 (0.01)

Heart failure     1.03 (0.02)

Uncomplicated hypertension     0.98 (0.01)

Hypertension with involvement target organs     0.99 (0.02)

Transient cerebral ischemia     0.90 (0.03)

Hemorrhoids     1.02 (0.03)

Rheumatoid arthritis and allied conditions     1.03 (0.03)

Osteoarthritis of hip     0.96 (0.04)

Osteoarthritis of knee     1.00 (0.03)

Other osteoarthritis and allied conditions     1.01 (0.04)

Osteoporosis     1.04 (0.03)

Headache     1.03 (0.02)

Tension headache     1.06 (0.04)

Restless legs syndrome     1.03 (0.04)

Vertigo/dizziness     0.97 (0.03)

Parkinsonism/paralysis agitans     1.02 (0.05)

Epilepsy, all types     1.06 (0.03)

Migraine     1.05 (0.02)

Disturbances of sleep/insomnia     0.97 (0.01)

Affective psychosis     1.12 (0.06)

Anxiety disorder/anxiety state     0.99 (0.02)

Depressive disorder/anxiety/depression     1.01 (0.01)

Cough     1.00 (0.01)

Sympt/complt sinus (incl. Pain)     1.08 (0.05)

U.R.I. (head cold)/rhinitis nos     1.00 (0.01)

Sinusitis acute/chronic     1.00 (0.01)

Tonsillitis acute     0.98 (0.03)

Acute laryngitis/tracheitis/croup     1.12 (0.05)

Acute bronchitis/bronchiolitis     1.18 (0.02)

Pneumonia     1.05 (0.03)

Chronic bronchitis/bronchiectasis     0.96 (0.02)

Emphysema/chronic obstructive pulmonary disease     1.01 (0.01)

Asthma     1.04 (0.01)

Hayfever, allergic rhinitis     1.01 (0.01)

Localised redness/erythema/rash of skin     1.11 (0.05)

Herpes zoster     1.03 (0.05)

Dermatophytosis     1.02 (0.01)

Moniliasis/monilia infection/candidiasis     1.05 (0.03)

Other infectious skin dis.nec/erysipelas     0.98 (0.03)

Impetigo     1.04 (0.03)

Seborrhoic dermatitis/other erythematous dermatoses     1.05 (0.02)

Atopic dermatitis/eczema     1.00 (0.02)

Contact dermatitis/skin allergy     0.99 (0.01)

Psoriasis w/wo arthropathy     1.04 (0.03)

Acne     1.05 (0.02)

Urticaria     1.05 (0.03)

Other disease skin/subcutaneous tissue     0.99 (0.04)

Hyperthyroidism/thyrotoxicosis     0.96 (0.04)

Hypothyroidism/myxedema     0.85 (0.02)

Diabetes mellitus     1.00 (0.01)

Gout     0.94 (0.03)

Lipid metabolism disorder     0.96 (0.01)

Cystitis/other urinary infect. Non-venereal     0.98 (0.01)

Family planning/oral contraceptive     1.03 (0.01)

Menstrual pain     0.98 (0.04)

Menstruation excessive     1.03 (0.04)

Menstruation irregular/frequent     1.05 (0.05)

Menopausal sympt./complt.     1.01 (0.02)

Urogenital candidiasis, thrush (proven/unproven)     1.03 (0.02)

Vaginitis/vulvitis, non venereal nos     1.03 (0.04)

Benign prostatic hypertrophy     1.00 (0.04)

Appendix 4 Total model used to measure the HHI

Fixed effects     Estimate (st. error)

DSS use:

DSS daily users     40.3 (1.17)

DSS non users/havers     41.4 (1.27)

EMR:

EMR1 (centered)     -0.37 (2.99)

EMR2 (centered)     -2.06 (3.15)

EMR3 (centered)     -3.53 (3.09)

Type of practice:

Single handed practice (centered)     10.9 (5.14)

Duo practice (centered)     9.23 (4.37)

Group practice (centered)     5.43 (2.90)

Dispensing practice (centered)     2.04 (3.16)

Number of GPs (centered)     2.07 (1.24)

Number of different drugs advised in the DSS (centered)     -3.37 (0.10)

Random effects     Variance (st. error)

Practice level:

DSS daily users     27.5 (9.35)

DSS non users/havers     0.00 (0.00)

GP level:

DSS daily users     3.48 (3.09)

DSS non users/havers     44.9 (10.9)

Diagnosis level:

Pain: generalized/unspecified     912.9 (153.8)

Allergy/allergic reaction nos     350.1 (55.69)

No disease     1,005 (206.3)

Iron deficiency anemia     1,500 (232.6)

Pernicious/folate deficiency anemia     1,302 (235.6)

Stomach ache/stomach pain     418.2 (65.43)

Heartburn     399.2 (62.86)

Nausea     979.8 (154.3)

Diarrhea     640 (102.4)

Constipation     233.3 (36.55)

Other presumed infections of digestive system     676.2 (112.6)

Disease of esophagus     804.6 (126)

Disorders of stomach function/gastritis     484.1 (76.6)

Irritable bowel syndrome     473.6 (74.17)

Anal fissure/perianal abscess     603.7 (102.1)

Abnormal sensations of eye     716.1 (114.9)

Infectious conjunctivitis     918.8 (140)

Allergic conjunctivitis     471.7 (73.22)

Otitis externa     470.5 (72.37)

Acute otitis media/myringitis     269.5 (42.23)

Vertiginous syndrome/labyrinthitis/vestibulitis     1125 (179.1)

Angina pectoris     402.9 (64.12)

Heart failure     273.9 (43.42)

Uncomplicated hypertension     398.7 (61.52)

Hypertension with involvement target organs     619.7 (100.6)

Transient cerebral ischemia     1,626 (269)

Hemorrhoids     438.1 (67.8)

Rheumatoid arthritis and allied conditions     543.6 (88.25)

Osteoarthritis of hip     942.2 (161)

Osteoarthritis of knee     315.7 (53.24)

Other osteoarthritis and allied conditions     851.8 (146.7)

Osteoporosis     556.7 (91.72)

Headache     233.8 (36.95)

Tension headache     438.8 (71.34)

Restless legs syndrome     1,141 (194.2)

Vertigo/dizziness     818.8 (128.2)

Parkinsonism/paralysis agitans     1,484 (278.3)

Epilepsy, all types     702.5 (116.1)

Migraine     767.8 (118)

Disturbances of sleep/insomnia     304.8 (47.83)

Affective psychosis     1,131 (233.7)

Anxiety disorder/anxiety state     297.3 (47.08)

Depressive disorder/anxiety/depression     194.7 (30.77)

Cough     621.1 (95.94)

Sympt/complt sinus (incl. Pain)     1465 (282.6)

U.R.I. (head cold)/rhinitis nos     837.8 (128.9)

Sinusitis acute/chronic     99.38 (15.89)

Tonsillitis acute     572.7 (89.45)

Acute laryngitis/tracheitis/croup     1,103 (188.4)

Acute bronchitis/bronchiolitis     1,058 (162.9)

Pneumonia     495.7 (77.23)

Chronic bronchitis/bronchiectasis     527.5 (86.3)

Emphysema/chronic obstructive pulmonary disease     57.73 (9.49)

Asthma     69.96 (11.34)

Hayfever, allergic rhinitis     300 (46.59)

Localised redness/erythema/rash of skin     676.7 (107.1)

Herpes zoster     736.8 (118.6)

Dermatophytosis     197.5 (30.83)

Moniliasis/monilia infection/candidiasis     592.3 (94.62)

Other infectious skin dis.nec/erysipelas     302.6 (48.2)

Impetigo     682.8 (106.5)

Seborrhoic dermatitis/other erythematous dermatoses     488.4 (75.41)

Atopic dermatitis/eczema     188.8 (29.58)

Contact dermatitis/skin allergy     352 (54.49)

Psoriasis w/wo arthropathy     276.2 (43.5)

Acne     180.6 (28.32)

Urticaria     482.5 (74.82)

Other disease skin/subcutaneous tissue     633 (99.3)

Hyperthyroidism/thyrotoxicosis     1,067 (190.4)

Hypothyroidism/myxedema     1,731 (269.9)

Diabetes mellitus     304.3 (47.23)

Gout     384.1 (60.24)

Lipid metabolism disorder     1,051 (161.1)

Cystitis/other urinary infect. Non-venereal     130.7 (20.6)

Family planning/oral contraceptive     354.2 (54.62)

Menstrual pain     669 (111.2)

Menstruation excessive     864.6 (135.5)

Menstruation irregular/frequent     1,708 (272.2)

Menopausal sympt./complt.     243.3 (37.94)

Urogenital candidiasis, thrush (proven/unproven)     193.9 (30.39)

Vaginitis/vulvitis, non venereal nos     659.5 (106.3)

Benign prostatic hypertrophy     1,616 (267.3)

## Pre-publication history

The pre-publication history for this paper can be accessed here:


